# Evaluation of the advantages of robotic versus laparoscopic surgery in elderly patients with colorectal cancer

**DOI:** 10.1186/s12877-023-03822-4

**Published:** 2023-02-20

**Authors:** Yonggan Xue, Sen Li, Shaohua Guo, Yanshen Kuang, Mu Ke, Xin Liu, Fangming Gong, Peng Li, Baoqing Jia

**Affiliations:** 1 Department of General Surgery, The First Medical Centre, Chinese PLA General Hospital, Haidian District, No.28, Fuxing Road, Beijing, 100853 China; 2 Department of General Surgery, The Eighth Medical Center, Chinese PLA General Hospital, Haidian District, No.Jia17, Heishanhu Road, Beijing, 100089 China

**Keywords:** Colorectal cancer, Elderly patients, Robotic surgery

## Abstract

**Background:**

The incidence of colorectal cancer increases with aging. Curative-intent surgery based on a minimally invasive concept is expected to bring survival benefits to elderly patients (aged over 80 years) with colorectal cancer who are frequently with fragile health status and advanced tumors. The study explored survival outcomes in this patient population who received robotic or laparoscopic surgery and aimed to identify an optimal surgical option for those patients.

**Methods:**

We retrieved the clinical materials and follow-up data on elderly patients with colorectal carcinoma who received robotic or laparoscopic surgery in our institution. The pathological and surgical outcomes were compared to examine the efficacy and safety of the two approaches. The DFS (disease-free survival) and OS (overall survival) results at 3 years after surgery were assessed to explore the survival benefits.

**Results:**

A total of 111 patients were screened for the study, including 55 in the robotic group and 56 in the laparoscopic group. The demographic details were generally similar between the two groups. No statistically significant difference in the number of removed lymph nodes was observed between the two approaches, with a median of 15 versus 14 (*P* = 0.053). The intraoperative blood loss was significantly reduced by robotic technique when compared to the laparoscopic approach, with a mean of 76.9 ml versus 161.6 ml (*P* = 0.025). There were no significant differences in operation time, conversion, postoperative complications and recovery, and long-term outcomes between the two groups.

**Conclusion:**

Robotic surgery was prized for elderly patients with colorectal cancer who developed anemia and/or hematological conditions.

**Supplementary Information:**

The online version contains supplementary material available at 10.1186/s12877-023-03822-4.

## Introduction

Colorectal cancer remains prevalent in the world. The incidence of colorectal cancer is expected to increase as the population ages [1, 2]. Thus, amid the rapid expansion of the elderly population, surgical operations in the fast-aging group are on the rise. Elderly patients (aged ≥ 80 years) are more likely to have a weakened functional status, frailty, and multiple comorbidities, which poses them with a higher risk of postoperative morbidities and mortality. Additionally, colorectal cancer in elderly patients is more advanced when diagnosed, partly due to the challenges in cancer surveillance and registration in this age group [3]. These conditions largely make surgeons hesitate to perform curative surgical treatment on elderly patients [4]. Notably, it has been reported that elderly cancer patients who survive 1 year can have a comparable 5-year survival outcome to that of middle-aged patients, highlighting the importance of conducting curative-intent aggressive treatment, including surgical intervention, for elderly patients [5].

Mounting evidence has confirmed the short-term advantages and long-term efficacy of laparoscopic surgery in the treatment of colorectal cancer when compared to traditional open surgery[6, 7]. More recent studies primarily focusing on laparoscopic technique for treating elderly patients with colorectal cancer have found that laparoscopic-assisted surgery offers several perioperative benefits and similar long-term survival outcomes as compared to open surgery, and is feasible and safe for a curative resection [8, 9].

Robotic-assisted surgery has increasingly gained acknowledgment in the field of colorectal carcinoma resection [10]. Robotic surgery system serves surgeons with advantageous instrument utilities, including immersive 3-dimensional magnified vision, flexible instruments, a stable camera, and ergonomic improvements, that practically circumvent limitations of conventical laparoscopic operation. Former studies have suggested that robotic colorectal cancer surgery yields short-term postoperative and oncological outcomes comparable to the laparoscopic approach [11, 12]. Notably, robotic assistance shows an advantage over conventional laparoscopic technique, with a reduced need to convert to open surgery, particularly, in the context of middle and low rectal carcinoma [13]. However, robotic surgery is typically correlated with longer operational time and higher hospital costs [14, 15]. Despite this, the increasing adoption of robotic resection to treat colorectal cancer is not compromised. Up to date, report on robotic advantage for treating fast-growing elderly patients with colorectal malignancy remains rare, warranting a need to assess the usefulness and potential expansion of robotic surgery in those patients.

Our team started to perform laparoscopic colorectal cancer surgery in 2008 and robotic colorectal cancer surgery the next year. With the relevant surgical experience accumulating, we began to attempt laparoscopic colorectal surgery for elderly patients in 2010 and robotic colorectal surgery for this age group patients in 2012. Until December 2021, we have accomplished 55 cases of robotic colorectal tumor resection and 56 cases of laparoscopic colorectal tumor resection for elderly patients aged 80 years or older with colorectal cancer. Here, we retrospectively reviewed the short-term and long-term outcomes of robotic versus laparoscopic colorectal cancer surgery in elderly patients and aimed to evaluate the feasibility and security of robotic and laparoscopic intervention in this patient population, as well as compared the survival outcomes of the two techniques. This article is expected to shed light on clinicians’ choice of optimal surgical approach for elderly patients with colorectal carcinomas.

## Methods

### Patients and surgical approaches

This is a retrospective, single-center study examining the efficacy and safety, as well as long-term outcomes of robotic versus laparoscopic surgery for elderly patients with colorectal cancer. All the patients were aged 80 years or older and underwent curative-intent surgery for colorectal carcinoma at the Chinese People's Liberation Army general (PLA) hospital. The patients or their relatives made the final decision to receive either robotic or laparoscopic surgery after the capacities of the two surgical systems were explained in detail to them by the surgeons. To avoid surgeon’s experience bias, we included the only patients who underwent surgeries completed by a single surgical team. All the surgical procedures were performed under the principles of total mesocolic excision (for colonic cancers) and total mesorectal excision (for rectal cancers). Written informed consent was provided by either the individual patients or their immediate family. The study was approved by Ethics Committee in the institution.

### Diagnosis and staging

For patients with colonic cancer, the examination of computed tomography (CT) of the chest, abdomen, and pelvis was routinely performed before surgery; for patients with rectal cancer, the examination of chest and abdomen CT, as well as magnetic resonance imaging (MRI) of the pelvis, was routinely performed before surgery. Preoperative diagnosis of colorectal cancer was determined by a senior pathologist examining endoscopic multiple biopsies. The stage of the disease was determined by postoperative pathological examination on surgical specimens as well as preoperative imaging including chest CT, abdomen CT and/or MRI, pelvis CT or MRI, and positron emission tomography-computed tomography (if necessary).

### Preoperative preparation

For patients who developed anemia, a blood transfusion was performed to ensure preoperative hemoglobin concentration was maintained at 9.0 g/dL or above. Patients with decreased albumin (albumin < 35 g/L) at admission routinely received a preoperative nutritional intervention. All patients underwent arterial blood gas analysis, pulmonary function test, cardiac function examination, and others, like cerebrovascular relevant examination (if necessary), after admission. Preoperative consultation involving a cardiologist, respiratory physician, and anesthesiologist was held for individual patients to exclude absolute surgical contraindications.

### Demographics, laboratory tests before surgery, and outcomes

Demographic characteristics, laboratory examination, and operative and postoperative outcomes were retrieved from the electric medical record system in our institution by a trained research assistant. Patients’ baseline demographics include age, gender, BMI (body mass index), ASA score (American Society of Anesthesiologists I-IV), previous abdomen surgery, comorbidity, synchronous tumor, ACCI (age-adjusted Charlson Comorbidity index), neoadjuvant therapy, tumor location, Serum CEA, Serum CA19-9, Serum CA724, and Serum CA125. To holistically evaluate the patient’s health condition prior to surgery, we drew on the relative indexes to reflect the underlying capacity of hematopoiesis, nutrition, liver, kidney, and lung to tolerate surgical intervention.

Surgical and postoperative short-term outcomes included surgical procedure, Anesthetic time, Surgical operative time, blood loss and transfusion, convention, the need for intensive care unit (ICU), multi-visceral resection, time to flatus, time to soft food intake, length of hospital stay, mortality within 30 days, morbidity within 30 days, and readmission within 30 days. Pathological staging was determined in accordance with the Union for International Cancer Control (UICC) 8th version. Pathological outcomes included tumor size, histologic type, resected lymph node status, TNM stage, vascular invasion, perineural invasion, circumferential resection margin (CRM), distal resection margin (DRM), and tumor residues.

The patients were followed up every 3 months during the first 3 years and then every 6 months till death or the end of October 2022. The examination of CT of the chest, abdomen, and pelvis as well as serum CEA level reflect disease progression. Disease-free survival was defined as the duration from the first day after surgery till the time a diagnosis of recurrence was identified. Overall survival was defined as the time from the first day after surgery till death or October 2022.

### End points

The study evaluated two primary endpoints, including the primary efficacy endpoint and the primary safety endpoint. The primary efficacy endpoint compared the pathological outcomes between the two groups. The primary safety endpoints compared the surgical and postoperative outcomes between the two groups. The key secondary endpoints included disease-free survival (DFS) and overall survival (OS) at 3-year after surgery between the two groups.

### Statistical analysis

Continuous variables were presented as median (IQR) or mean (SD). Continuous variables adhering to normal distribution were analyzed with the Student’s T-test, whereas non-normal distribution ones were evaluated with the Mann–Whitney U test. Categorical variables were shown with numbers and percentages and were compared with the chi-squared test or Fisher’s exact test. Long-term outcomes were calculated using the Kaplan–Meier method, and log-rank tests were employed to compare differences between the groups. *P* value < 0.050 was considered a statistically significant difference. All analyses were performed SPSS software (version 27.0.1.0).

## Results

### Patients

From March 1, 2010, till December 27, 2021, a total of 111 elderly patients with colorectal cancer who received minimally invasive surgery were screened for the study. Of those patients, 55 underwent robotic surgery (robotic group), and 56 experienced laparoscopic surgery (laparoscopic group) (Supplementary Figure). The demographic characteristics of the patients were generally similar between the two groups (Table 1). The patients in the robotic group tend to rate a higher ASA score than those in the laparoscopic group. The laparoscopic group had more patients experiencing prior abdominal surgery than the robotic group (*P* < 0.01), but the comorbidity of the patients at the time point of the colorectal surgery showed a converse tendency between the two groups (*P* = 0.01). 4 patients in the robotic group had a synchronous tumor, while the laparoscopic had 5. 2 patients received neoadjuvant therapy and underwent robotic surgery. The levels of serum cancer biomarkers, including CEA, CA19-9, CA724, and CA125 at admission, were no significant difference between the two groups. There were no evident differences in age, gender, BMI, ACCI, and tumor location between the two groups.

**Table 1:** Demographic characteristics of the patients at baseline

	**Robotic surgery (*n* = 55)**	**Laparoscopic surgery (*n* = 56)**	**Overall (*n* = 111)**	**P**
Age, median (IQR), years	82(81–85)	82(81–84)	82(81–85)	0.202
Gender, n (%)				0.294
Male	31(56.4)	37(66.1)	68(61.3)	
Female	24(43.6)	19(33.9)	43(38.7)	
BMI, mean (SD), kg/m^2^	23.8(3.6)	23.3(2.7)	23.5(3.2)	0.362
ASA score, n (%)				0.782
II	26(47.3)	30(53.6)	56(50.6)	
III	28(50.9)	25(44.6)	53(47.7)	
IV	1(1.8)	1(1.8)	2(1.8)	
Previous abdominal surgery, n (%)	10(18.2)	24(42.9)	34(30.6)	0.005
Hepatobiliary surgery	5(9.1)	6(10.7)	11(9.9)	
Gynecological surgery	2(3.6)	7(12.5)	9(8.1)	
Appendectomy	4(7.3)	11(19.6)	15(13.5)	
Anterior resection	0(0)	1(1.8)	1(0.9)	
Inguinal herniorrhaphy	0(0)	2(3.6)	2(1.8)	
Urological surgery	1(1.8)	1(1.8)	2(1.8)	
Comorbidity, n (%)	44(80.0)	32(57.1)	76(68.5)	0.010
Coronary heart disease	12(21.8)	11(19.6)	23(20.7)	
Diabetes	12(21.8)	6(10.7)	18(16.2)	
Cerebrovascular disease	10(18.2)	6(10.7)	16(14.4)	
Hepatitis	2(3.6)	2(3.6)	4(3.6)	
Renal disease	4(7.3)	2(3.6)	6(5.4)	
Hypertension	30(54.5)	27(48.2)	57(51.4)	
Pulmonary disease	4(7.3)	3(5.4)	7(7.6)	
Synchronous tumor^*^, n (%)	4(7.3)	5(8.9)	9(8.1)	> 0.999
ACCI, mean (SD)	7.1(1.1)	7.2(1.8)	7.2(1.5)	0.505
Neoadjuvant therapy, n (%)	2(3.6)	0(0)	2(1.8)	0.243
Tumor location, n (%)				0.391
Ascending colon	16(29.1)	19(33.9)	35(31.5)	
Transverse colon	0(0)	2(3.6)	2(1.8)	
Descending colon	2(3.6)	1(1.8)	3(2.7)	
Sigmoid colon	13(23.6)	16(28.6)	29(26.1)	
Rectum	22(40.0)	15(26.8)	37(33.3)	
Other^**^	2(3.6)	3(5.4)	5(4.5)	
Serum CEA, median (IQR), μg/L	4.4(2.6–9.9)	7.3(3.3–20.8)	5.5(2.9–13.4)	0.754
Missing data, n (%)	0(0)	1(1.8)	1(0.9)	
Serum CA19-9, median (IQR), u/mL	14.3(7.8–20.5)	18.9(8.9–42.6)	15.8(8.6–29.4)	0.079
Missing data, n (%)	0(0)	1(1.8)	1(0.9)	
Serum CA724, median (IQR), u/mL	2.7(1.6–5.2)	2.3(1.4–6.6)	2.6(1.4–5.3)	0.805
Missing data, n (%)	3(5.5)	5(8.9)	8(7.2)	
Serum CA125, median (IQR), u/mL	11.3(8.0–18.8)	12.4(8.8–16.3)	11.6(8.4–18.1)	0.803
Missing data, n (%)	1(1.8)	3(5.4)	4(3.6)	

Abbreviation: *IQR* Interquartile range, *BMI* Body mass index, *SD* Standard deviation, *ASA* American Society of Anesthesiologists, *ACCI* Age-adjusted Charlson Comorbidity Index, *CEA* Carcinoembryonic antigen, *CA* Carbohydrate antigen

^*^The robotic group has 2 patients with synchronic liver metastasis, 1 patient with synchronic lung metastasis, and 1 with synchronic ovary metastasis. The laparoscopic group has 4 patients with synchronic liver metastasis and 1 with synchronic ovary metastasis

^**^The robotic group has 1 patient who developed ascending colon tumor and rectal tumor simultaneously at admission, 1 patient who developed ascending colon tumor and sigmoid tumor. The laparoscopic group has 1 patient who developed ascending colon tumor and rectal tumor simultaneously at admission, 1 patient who developed ascending colon tumor and sigmoid tumor, and 1 patient who developed sigmoid tumor and rectal tumor

The laboratory test results, mostly reflecting hematopoietic function, nutritional condition, hepatic and renal function, and cardio-pulmonary function, were generally balanced at admission between the two groups, apart from the median levels of serum total protein being lower in the robotic group as compared with the laparoscopic group (Supplementary Table).

### Efficacy

The pathological outcomes of patients after surgery were retrieved and used to compare the efficacy of the two surgical approaches (Table 2). No statistically significant difference in the number of resected lymph nodes was observed between robotic versus laparoscopic surgery, with a median lymph node yield of 15 and 14 in the respective group (*P* = 0.053). Notably, the laparoscopic group had a significantly higher percentage of patients with positive lymph nodes (lymphatic metastasis) than the robotic group, 51.8% versus 30.9% (*P* = 0.026). As a consequence, more patients in the laparoscopic group were defined into higher pathological N stage than those in the robotic group, with the pN1 stage finding 41.1% versus 27.3% and the pN2 stage finding 14.3% versus 9.1%, although the differences did not reach significance. The laparoscopic group tended to have a higher percentage of patients identified with more advanced tumors by T stage compared with the robotic group. Consistently, a higher proportion of patients in the laparoscopic group appeared to have tumors graded at a high TNM stage compared with that in the robotic group, particularly for stage III tumors. Therefore, the patients in the laparoscopic group had a higher degree of cancer progression in comparison to the robotic group. There were no significant differences in tumor size, histological type, vascular invasion, perineural invasion, CRM, and DRM between the two groups. All patients achieved R0 resection in both groups. Collectively, no significant difference was identified between the two approaches when it comes to the efficacy of surgery.

**Table 2:** Pathological outcomes of the patients

	**Robotic surgery (*n* = 55)**	**Laparoscopic surgery (*n* = 56)**	**Overall (*n* = 111)**	**P**
Tumor size, mean (SD), cm	4.9(2.3)	4.5(2.1)	4.7(2.2)	0.279
Histologic type, n (%)				0.476
Well	1(1.8)	0(0)	1(0.9)	
Moderate	45(81.8)	43(76.8)	88(79.3)	
Poor	9(16.4)	13(23.2)	22(19.8)	
Number of resected LN, median (IQR)	15(12–20)	14(9.3–14.8)	15(11–18)	0.053
Proportion of positive LN, n (%)	17(30.9)	29(51.8)	46(41.4)	0.026
T stage, n (%)				0.575
pT1	3(5.5)	2(3.6)	5(4.5)	
pT2	7(12.7)	7(12.5)	14(12.6)	
pT3	37(67.3)	33(58.9)	70(63.1)	
pT4	8(14.5)	14(25.0)	22(19.8)	
N stage^*^, n (%)				0.133
pN0	35(63.6)	25(44.6)	70(63.1)	
pN1	15(27.3)	23(41.1)	38(34.2)	
pN2	5(9.1)	8(14.3)	13(11.7)	
TNM stage^**^, n (%)				0.353
I	7(12.7)	5(8.9)	12(10.8)	
II	26(47.3)	19(33.9)	45(40.5)	
III	18(32.7)	27(48.2)	45(40.5)	
IV	4(7.3)	5(8.9)	9(8.1)	
Vascular invasion, n (%)	9(16.4)	6(10.7)	15(13.5)	0.384
Perineural invasion, n (%)	3(5.5)	7(12.5)	10(9.0)	0.321
CRM-positive, n (%)	1(1.8)	0(0)	1(0.9)	0.495
DRM-positive, n (%)	0(0)	0(0)	0(0)	N/A
Residual tumor, n (%)				N/A
R0	55(100.0)	56(100.0)	111(100.0)	
R1	0(0)	0(0)	0(0)	

Abbreviation: *SD* Standard deviation, *LN* lymph nodes, *IQR* Interquartile range, *CRM* Circumferential resection margin, *DRM* Distal resection margin

^*^Tumor deposit was counted as N1c according to the UICC 8th version

^**^The TNM stage was determined according to the more advanced tumor if a patient simultaneously developed two colorectal tumors

### Safety

The surgical and postoperative outcomes were employed to evaluate the safety of the two surgical techniques (Table 3). The surgical procedure was comparable between the groups. Intraoperative blood loss in robotic surgery was statistically significantly less than that in laparoscopic surgery, with a mean intraoperative blood loss of 76.9 ml in the robotic group and 161.6 ml in the laparoscopic group, respectively (*P* = 0.025). 5 patients in the robotic group received blood transfusion during or after surgery, but 11 did in the laparoscopic group. 1 patient in the robotic group experienced conversion, while no patient in the laparoscopic group did that. There were no significant differences in anesthetic time, surgical operative time, ICU need, and ICU stay between the two groups. Postoperative outcomes, including time to flatus, soft food intake, postoperative hospital stay, morbidity and readmission within 30 days after surgery, and Clavien-Dindo classification, were generally similar between the two groups. No hospital death occurred within 30 days after surgery in both groups.

**Table 3:** Surgical outcomes and postoperative outcomes of the patients

	**Robotic surgery (*n* = 55)**	**Laparoscopic surgery (*n* = 56)**	**Overall (*n* = 111)**	**P**
Surgical procedure, n (%)				0.152
Radical right hemicolectomy	17(30.9)	20(35.7)	37(33.3)	
Transverse colectomy	0(0)	2(3.6)	2(1.8)	
Radical left hemicolectomy	3(5.5)	1(1.8)	4(3.6)	
Sigmoid colectomy	12(21.8)	15(26.8)	27(24.3)	
Anterior resection	21(38.2)	11(19.6)	32(28.8)	
Abdominoperineal resection	1(1.8)	4(7.1)	5(4.5)	
Other^*^	1(1.8)	3(5.4)	4(3.6)	
Anesthetic time, mean (SD), min	230.7(56.4)	223.6(62.2)	227.1(59.2)	0.531
Surgical operative time, mean (SD), min	174.4(52.3)	170.2(58.8)	172.3(55.5)	0.690
Blood loss, mean (SD), mL	76.9(39.7)	161.6(207.1)	119.6(155.0)	0.025
Transfusion, n (%)	5(9.1)	11(19.6)	16(14.4)	0.114
Conversion, n (%)	1(1.8)	0(0)	1(0.9)	0.495
ICU need, n (%)	17(30.9)	15(26.8)	32(28.8)	0.632
ICU stay, median (IQR), days	0(0–1)	0(0–1)	0(0–1)	0.629
Multivisceral resection, n (%)	2(3.6)	3(5.4)	5(4.5)	> 0.999
Time to flatus, median (IQR), days	2(2–4)	3(2–4)	3(2–4)	0.330
Time to soft food intake, median (IQR), days	4(4–5)	4.5(4–6)	4(4–6)	0.080
Length of postoperative hospital stay, median (IQR), days	10(8–12)	9(8–11)	10(8–12)	0.795
Mortality within 30 days, n (%)	0(0)	0(0)	0(0)	N/A
Morbidity within 30 days, n (%)	10(18.2)	12(21.4)	22(19.8)	0.668
Anastomotic leakage	1(1.8)	1(1.8)	2(1.8)	
Cardiovascular event	4(7.3)	6(10.7)	10(9.0)	
Postoperative ileus	1(1.8)	2(3.6)	3(2.7)	
Pneumonia	1(1.8)	1(1.8)	2(1.8)	
Urinary retention	1(1.8)	2(3.6)	3(2.7)	
Abdominal/anastomotic bleeding	1(1.8)	0(0)	1(0.9)	
Wound infection	1(1.8)	1(1.8)	2(1.8)	
Delirium	1(1.8)	0(0)	1(0.9)	
Abdominal abscess	1(1.8)	1(1.8)	2(1.8)	
Lymphatic fistula	1(1.8)	0(0)	1(0.9)	
Rectovaginal fistula	1(1.8)	0(0)	1(0.9)	
Clavien-Dindo classification, n (%)				0.444
II	7(12.7)	10(17.9)	17(15.3)	
III	1(1.8)	0(0)	1(0.9)	
IV	0(0)	0(0)	0(0)	
V	0(0)	0(0)	0(0)	
Readmission within 30 days, n (%)	1(1.8)	1(1.8)	2(1.8)	> 0.999

Abbreviation: *SD* Standard deviation, *ICU* Intensive care unit, *IQR* Interquartile range

^*^The robotic group has 1 patient who received right colon and sigmoid colon radical surgery. The laparoscopic group has 1 patient who underwent right colon and sigmoid colon radical surgery, 1 right colon and rectum radical surgery, and 1 sigmoid colon and rectum radical surgery

### Survival outcomes

**Fig. 1:**
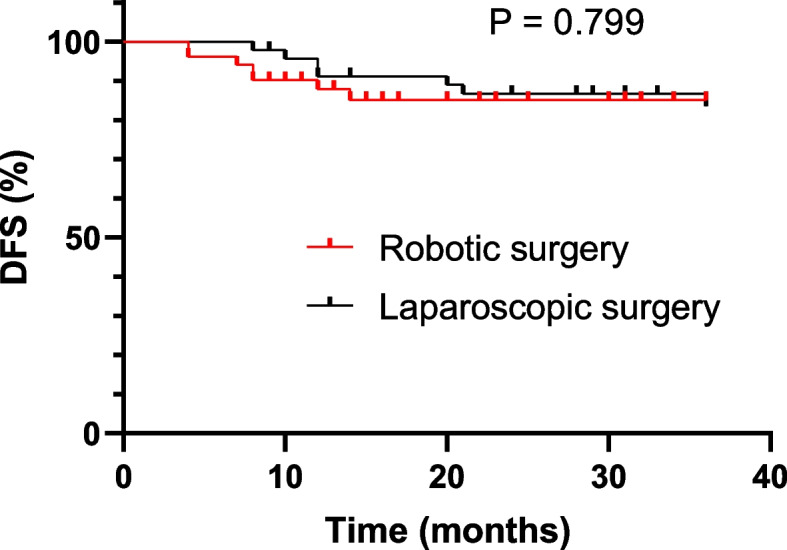
DFS of robotic versus laparoscopic surgery at 3-year after surgery

**Fig. 2:**
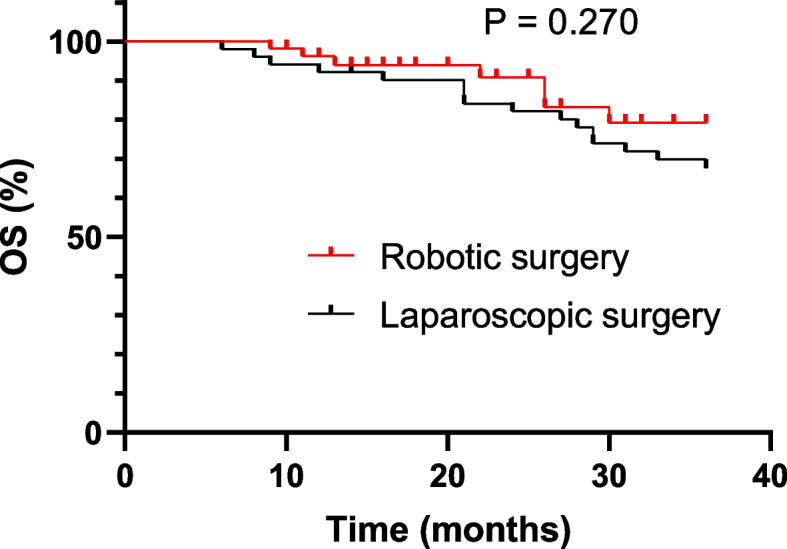
OS of robotic versus laparoscopic surgery at 3-year after surgery

Four patients in the robotic group and 5 patients in the laparoscopic group were diagnosed with a metastatic disease before surgery, which was omitted from DFS analyses. 5 patients, including 1 patient who developed metastatic disease before surgery, in the laparoscopic group were lost to the follow-up and, thus, omitted from the OS analyses. The median duration follow-up was 23 (IQR, 12 to 44) months in the robotic group and 44 (IQR, 29 to 71) months in the laparoscopic group. During the period of follow-up, 7 patients in the robotic group developed disease relapse, including 2 patients with local recurrence, 2 pulmonary metastasis, 2 multi-organ metastasis, and 1 hepatic metastasis (Table 4). 13 patients in the laparoscopic group experienced disease relapse, including 7 patients with multi-organ metastasis, 3 hepatic metastasis, 2 pulmonary metastasis, and 1 local recurrence (Table 4). The 3-year DFS outcomes were similar between the two groups, with the robotic group achieving 85.1% and the laparoscopic group 83.5% (Fig. 1). Similarly, the 3-year OS outcomes were of no statistically significant difference between the two groups, 79.3% versus 67.6% in the respective group (Fig. 2).

**Table 4:** Details in recurrence of the patients

	**Robotic surgery (*n* = 51)**	**Laparoscopic surgery (*n* = 47)**	**Overall (*n* = 98)**	**P**
Duration of surveillance, median (IQR), months	23 (12–44)	44 (29–71)	33(18–63)	N/A
Overall recurrence, n (%)	7(13.7)	13(27.7)	20(20.4)	0.087
Primary tumor site	2(3.9)	1(2.1)	3(3.1)	
Liver	1(2.0)	3(6.4)	4(4.1)	
Lung	2(3.9)	2(4.2)	3(3.1)	
Multiple organs	2(3.9)	7(14.9)	9(9.2)	

Abbreviation: *IQR* Interquartile range

During the period of surveillance, 7 patients in the robotic group were dead, with 4 dead of the primary tumor and 3 other causes including 1 cerebrovascular disease and 2 unknowns (Table 5). In the laparoscopic group, 13 patients were found dead, including 3 dead of the primary tumor and 10 other conditions consisting of 1 cerebrovascular disease, 3 cardiovascular diseases, 1 MODS, 1 colitis with bleeding, 2 other tumors, and 2 unknowns (Table 5).

**Table 5:** Details in death of the patients

	**Robotic surgery (*n* = 51)**	**Laparoscopic surgery (*n* = 47)**	**Overall (*n* = 98)**
Primary tumor, n (%)	4(7.8)	3(6.4)	7(7.1)
Other diseases, n (%)	3(5.9)	10(21.3)	13(13.3)
Cerebrovascular disease	1(2.0)	1(2.1)	2(2.0)
Cardiovascular disease	0(0)	3(6.4)	3(3.1)
MODS	0(0)	1(2.1)	1(1.0)
Ulcer bleeding	0(0)	1(2.1)	1(1.0)
Other tumors	0(0)	2(4.3)	2(2.0)
Unknown	2(3.9)	2(4.3)	4(4.1)

Abbreviations: *MODS* Multiple organ dysfunction syndrome

## Discussion

To our best knowledge, this is the first report into comprehensively comparing the efficacy and safety, as well as long-term survival outcomes, of robotic surgery with laparoscopic surgery in managing elderly patients (aged 80 years or more) with colorectal cancer. Our findings revealed that both approaches can efficaciously excise tumors from this age group with equivalent pathological outcomes. The robotic technique significantly reduced intraoperative blood loss when compared with the laparoscopic approach. No hospital death occurred within 30 days after surgery in both groups, indicating the safety of the two approaches in treating elderly patients with colorectal cancer. In addition, long-term follow-up results demonstrated that elderly patients derived benefits from minimally invasive surgical intervention.

Considering the increased comorbidities, decreased resilience to surgical stress, and vulnerability to poor nutrition of elderly patients, clinicians tend to choose conservative treatment, like chemotherapy, palliative therapy, or even only symptomatic treatment, instead of curative-intent surgery [16]. In fact, this frequently leads to the undertreatment of this patient population [17]. Recent studies have revealed that in the context of colorectal cancer, elderly patients derived equivalent oncologic outcomes to younger patients after radical surgery and concluded that age was not an independent prognostic factor for long-term survival [18, 19]. Therefore, here is a pressing need to prompt suitable treatment options, including establishing an optimal surgical approach, for this age group.

Previous studies have revealed that laparoscopic surgery shows evident advantages over conventional open surgery with less surgical trauma, rapid recovery, and comparable survival outcomes, regardless of the age group. For elderly patients with colorectal cancer, laparoscopic surgery significantly reduced the postoperative complications and non-cancer-related death compared to open surgery [20]. A recent meta-analysis revealed that elderly patients derived apparent benefits from laparoscopic surgery, including less blood loss and shorten hospital stay, as well as equivalent long-term outcomes when compared with open surgery [8]. By virtue of a stable camera, articulated arms, and 3-dimensional scope, the robotic device has been increasingly introduced to the treatment of colorectal cancer and is expected to yield more favorable outcomes compared to the laparoscopic approach, which prompted us to access the practical application value of robotic surgery in elderly patients.

The present study revealed that both surgical approaches achieved the goal of completely removing the primary tumor, along with an adequate number of lymph node dissections. There was no statistically significant difference with regard to lymph node resection by robotic versus laparoscopic surgery, with a median number of lymph nodes resected of 15 in the robotic group versus 14 in the laparoscopic group (*P* = 0.053). Notably, we found that patients in the laparoscopic group had a higher proportion of lymph node metastasis, as compared with those in the robotic group, 51.8% versus 30.9% in the respective group. This is largely due to the fact that the laparoscopic group tended to have more advanced tumors compared to the robotic group before surgery, as evidenced by the tendency of postoperative pathological T stage and TNM stage. The two surgical approaches were comparable in terms of other pathological parameters, including vascular and perineural invasion, CRM and DRM status, and R stage.

Additionally, we found that compared to laparoscopic surgery, robotic surgery significantly reduced intraoperative blood loss, with a mean of 76.9 ml versus 161.6 ml, respectively (*P* = 0.025). This result was consistent with some previous observations in which robotic surgery contributed to less estimated blood loss when compared to laparoscopic approach in the treatment of colorectal cancer[13, 15]. This finding has the potential to guide clinicians to make choice of surgical approaches for patients, particularly important for those who developed anemia and/or coagulation dysfunction before surgery. A majority of studies reported that robotic surgery costs more time than laparoscopic surgery when treating patients with colorectal cancer [15, 21]. However, the present study did not show a significant difference in mean operative time between the groups, 174.4 min versus 170.2 min, respectively. This is partly because our team had accumulated adequate surgical skills when performing robotic surgery for the patients. Another factor might be that the patients in the laparoscopic group tended to have more advanced tumors and, therefore, needed longer time to complete a curative surgery. Although several studies reported that robotic surgical system reduced the probability of conversion when compared to conventional laparoscopic surgery [22, 23], there were still trials that did not show this advantage, even in patients with middle-low sites of rectal cancer [21, 24]. In the present study, only one patient in the robotic group underwent conversion, who was an 83-year-old female and suffered colonic obstruction due to the large tumor of 6 cm in diameter localized in the transverse colon near the hepatic flexure. The diffuse intestinal dilation heavily impeded the operation and, ultimately, led to the conversion. There was no conversion occurred in the laparoscopic group. Thus, we believed that the conversion event can be largely avoided for a well-skilled surgical team, irrespective of age group.

The complication rates within 30 days after surgery were similar between the groups, with 18.2% of patients in the robotic group and 21.4% in the laparoscopic group experiencing surgery-related events, which were in consistent with other studies [22, 25]. The most common complication was cardiovascular events in both groups, which was due to aging and heart conditions. The time to first flatus and soft food intake was comparable between the groups, and those time points were generally in line with other reports. Although the median length of hospital stay was no different between the two groups (10d versus 9d), this was longer than that in previous studies mainly focusing on younger age groups. This may reflect the slower recovery following surgery in the elderly population.

The long-term outcomes, including DFS and OS at 3 years after surgery were no significant difference between the groups. The 3-year DFS was 85.1% in the robotic group and 83.5% in the laparoscopic group. Robotic surgery yielded a 3-year OS of 79.3% and laparoscopic surgery 67.9%. These findings aligned with studies from other groups on elderly patients with colorectal cancer who received curative surgery [26].

Apparently, this study has limitations. First, although the sample number was generally matched between the two groups, the sample size was small, which required large-scale, multi-center studies on this topic. Moreover, while the basic demographic details were generally matched between the groups, the laparoscopic group tended to be pathologically confirmed with more advanced tumors. This would probably have impacts on surgical operations and long-term outcomes, which highlighted an unmet need for the design of studies pointing to the disease stage-based investigation. Additionally, another factor that should not be overlooked is the fact that the team started performing robotic surgery after completing certain cases of laparoscopic surgery in elderly patients involved. This not simply means that before conducting robotic surgery on the patients, the team already gained experience on the basis of laparoscopic surgery, but the patients in the robotic group had a relatively short period of follow-up. Future studies are expected to consider these factors.

In conclusion, this study comprehensively compared the efficacy and safety, along with survival outcomes, of robotic versus laparoscopic surgery in elderly patients with colorectal cancer. There were no significant differences in tumor-resecting efficiency, postoperative recovery, and long-term outcomes. Notably, the robotic approach significantly reduced intraoperative blood loss, as compared to laparoscopic surgery. These findings have the potential to guide clinicians’ choice of surgical approach for elderly patients with colorectal cancer, particularly for those with hematological conditions.

## Supplementary Information

**Additional file 1:** 

**Additional file 2:** 

## Data Availability

The datasets supporting the conclusions of this article are included within the article and its additional files.

## Abbreviations

ACCI: Age-adjusted Charlson Comorbidity Index
ALT: Alanine transaminase
ASA: American Society of Anesthesiologists
AST: Aspartate aminotransferase
BMI: Body mass index
CA: Carbohydrate antigen
CEA: Carcinoembryonic antigen
CRM: Circumferential resection margin
CT: Computed tomography
DFS: Disease-free survival
DRM: Distal resection margin
ICU: Intensive care unit
IQR: Interquartile range
LN: Lymph nodes
MODS: Multiple organ dysfunction syndrome
MRI: Magnetic resonance imaging
OS: Overall survival
PH: Pondus Hydrogenii
PLA: People's Liberation Army general
SD: Standard deviation
UICC: Union for International Cancer Control
