# Supplementary figures and images for: Evaluation of the advantages of robotic versus laparoscopic surgery in elderly patients with colorectal cancer
Source: BMC Geriatr. 2023 Feb 20;23:105. doi: 10.1186/s12877-023-03822-4 (PMC9942364; doi:10.1186/s12877-023-03822-4)

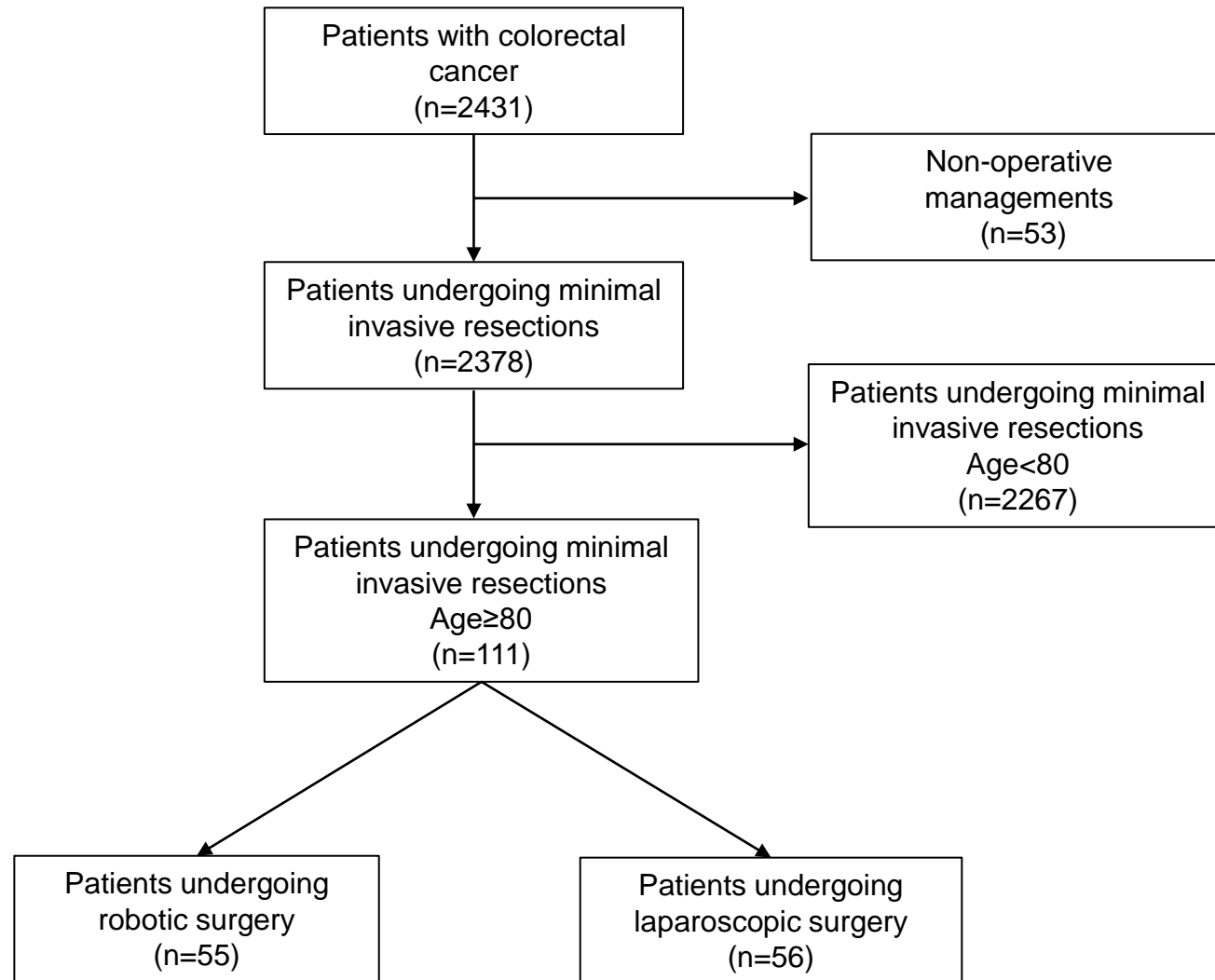

Supplementary Figure. Flowchart of patient selection.

Supplement: Supplementary file 1 — Additional file 1: [file 12877_2023_3822_MOESM1_ESM.pdf]
