# Supplementary material for: Evaluation of the advantages of robotic versus laparoscopic surgery in elderly patients with colorectal cancer
Source: BMC Geriatr. 2023 Feb 20;23:105. doi: 10.1186/s12877-023-03822-4 (PMC9942364; doi:10.1186/s12877-023-03822-4)
Supplement: Supplementary file 2 — Additional file 2: [file 12877_2023_3822_MOESM2_ESM.docx]

Supplementary Table. Lab examination prior to surgery.

|  | **Robotic surgery**  **(n=55)** | **Laparoscopic surgery**  **(n=56)** | **Overall**  **(n=111)** | **Reference value** | **P** |
| --- | --- | --- | --- | --- | --- |
| WBC, median (IQR), 10^12^/L | 5.4(4.5-6.5) | 5.8(4.9-6.7) | 5.6(4.6-6.7) | 3.5-10 | 0.280 |
| RBC, median (IQR), 10^9^/L | 3.9(3.6-4.2) | 4.1(3.7-4.4) | 4.0(3.7-4.3) | 4.3-5.9(Male)  2.9-5.2(Female) | 0.067 |
| Platelet, median (IQR), 10^9^/L | 195(166-236) | 211(154.3-247.8) | 204(161-241) | 100-300 | 0.737 |
| Hemoglobin, median (IQR), g/L | 111(102-124) | 118(102.5-131.5) | 115(102-128) | 137-179(Male)  116-155(Female) | 0.290 |
| Serum total protein, median (IQR), g/L | 62.4(58.0-65.3) | 64.6(61.0-68.8) | 63.6(59.5-66.6) | 55-80 | 0.003 |
| Serum albumin, median (IQR), g/L | 37.1(35.0-39.4) | 37.3(35.3-40.5) | 37.3(35.2-39.6) | 35-50 | 0.316 |
| Serum AST, median (IQR), u/L | 15.5(13.4-19.5) | 15.8(13.5-19.1) | 15.6(13.4-19.4) | 0-40 | 0.748 |
| Serum ALT, median (IQR), u/L | 10.4(8.6-15.4) | 10.4(8.5-14.1) | 10.4(8.6-14.1) | 0-40 | 0.984 |
| Serum total bilirubin, median (IQR), umol/L | 9.0(6.7-12.7) | 9.3(6.7-10.7) | 9.0(6.7-11.3) | 0-20 | 0.966 |
| Serum direct bilirubin, median (IQR), umol/L | 3.5(2.5-4.6) | 2.9(2.3-3.8) | 3.2(2.4-4.0) | 0-8.6 | 0.168 |
| Serum creatinine, median (IQR), umol/L | 75.4(61.2-86.1) | 75.9(67.6-95.6) | 75.8(65.5-89.2) | 30-110 | 0.335 |
| Blood Urea, median (IQR), mmol/L | 5.2(4.3-6.2) | 5.2(4.4-6.3) | 5.2(4.4-6.3) | 1.8-7.5 | 0.589 |
| Creatine kinase, median (IQR), u/L | 48.2(36.1-69.4) | 53.7(34.4-76.3) | 51.9(34.8-70.3) | 2-200 | 0.996 |
| Lactate dehydrogenase, median (IQR), u/L | 150.7(140.3-181.9) | 155.0(140.0-183.2) | 154.5(140.3-181.9) | 40-250 | 0.786 |
| PH value, median (IQR) | 7.37(7.35-7.39) | 7.37(7.34-7.39) | 7.37(7.34-7.39) | 7.35-7.45 | 0.037 |
| Missing Date, n (%) | 1(1.8) | 4(7.1) | 5(4.5) |  |  |
| Pa O_2_, mmHg | 86.7(79.7-101.7) | 83.3(77.5-92.0) | 84.5(78.1-94.9) | 80-100 | 0.133 |
| Missing Date, n (%) | 1(1.8) | 4(7.1) | 5(4.5) |  |  |
| Pa CO_2_, mmHg | 43.7(41.7-47.7) | 42.3(40.3-46.0) | 43.1(40.8-47.0) | 35-45 | 0.115 |
| Missing Date, n (%) | 1(1.8) | 4(7.1) | 5(4.5) |  |  |

Abbreviation: WBC, White blood cell; IQR, Interquartile range; RBC, Red blood cell; AST, Aspartate aminotransferase; ALT, Alanine transaminase; PH, Pondus Hydrogenii.
